# Neurosyphilis as a great imitator: a case report

**DOI:** 10.1186/s13104-016-2176-2

**Published:** 2016-07-28

**Authors:** Liis Sabre, Mark Braschinsky, Pille Taba

**Affiliations:** 1 Department of Neurology, Neurology Clinic, Tartu University Hospital, 8 L. Puusepa Street, 51014 Tartu, Estonia; 2 Department of Neurology and Neurosurgery, University of Tartu, Tartu, Estonia

**Keywords:** Movement disorders, Dementia, Neurosyphilis

## Abstract

**Background:**

Neurosyphilis is defined as any involvement of the central nervous system by the bacterium *Treponema pallidum*. Movement disorders as manifestations of syphilis have been reported quite rarely.

**Case presentation:**

We report a case of a 42-year-old Russian man living in Estonia with rapidly progressive dementia and movement disorders manifesting as myoclonus, cerebellar ataxia and parkinsonism. The mini mental state examination score was 12/30. After excluding different neurodegenerative causes, further diagnostic testing was consistent with neurosyphilis. Treatment with penicillin was started and 6 months later his mini mental state examination score was 25/30 and he had no myoclonus, parkinsonism or cerebellar dysfunction.

**Conclusion:**

Since syphilis is easily diagnosed and treatable, it should be considered and tested in patients with cognitive impairment and movement disorders.

**Electronic supplementary material:**

The online version of this article (doi:10.1186/s13104-016-2176-2) contains supplementary material, which is available to authorized users.

## Background

Neurosyphilis is defined as any involvement of the central nervous system by the bacterium *Treponema pallidum*. The annual incidence of neurosyphilis varies from 0.16 to 2.1 per 100,000 population [1]. It may involve the central nervous system at any stage of syphilitic infection. Early neurosyphilis manifests as meningitis, meningovascular syphilis or asymptomatic neurosyphilis [2]. Late neurosyphilis usually affects the brain and spinal cord, presenting as paretic neurosyphilis (general paresis) with neuropsychiatric manifestations including dementia, or tabetic neurosyphilis (*tabes dorsalis*) characterised by sensory ataxia, peripheral neuropathy and cranial nerve lesions. Movement disorders as manifestations of syphilis have been reported quite rarely [3, 4].

Here we describe a case of neurosyphilis presenting with parkinsonism, myoclonus, cerebellar ataxia and rapidly progressive dementia whose neurologic condition greatly improved after the antibiotic treatment with penicillin-G.

## Case presentation

A 42-year-old previously healthy Russian man living in Estonia was hospitalised due to a one-year history of progressive cognitive decline, confusion attacks, rare hallucinations, gait disturbances and involuntary movements.

At the beginning of the symptoms he was often sent on short sick leaves because of his employer’s doubt about his health. He resigned 6 months after the first symptoms appeared, being unable to perform his duties at work.

In addition, his spouse could not allow him to leave home due to his progressive disorientation. The patient became unable to cope with daily activities like dressing, brushing teeth and washing. He soon became dependent in most daily life activities. Therewith, he came to the neurologist for the first time.

On neurological examination he had predominantly left-sided bradykinesia and rigidity and intentional tremor in his left hand and both legs. There was no weakness on motor examination. Deep tendon reflexes were brisk and more pronounced in the left but there were no extensor reflexes. His gate was cautious and wide-based. There were myoclonic jerks in his legs and left arm that were more pronounced in action (stimulus-sensitive) (Additional file 1). According to the neuropsychological testing he had severe dementia with the mini mental state examination (MMSE) Score 12/30. The results were affected by severe attention deficit. He had affective symptoms like irritability, aggressive behaviour and delusions. His speech was dysarthric and dysphonic with mixed aphasia that included difficulties in word finding, impaired articulatory agility, verbal stereotypes, some paraphasias in running speech and difficulties in understanding longer sentences. He had anisocoria (the left pupil was larger), with pupils nonreactive to light, and horizontal nystagmus.

**Fig. 1:**
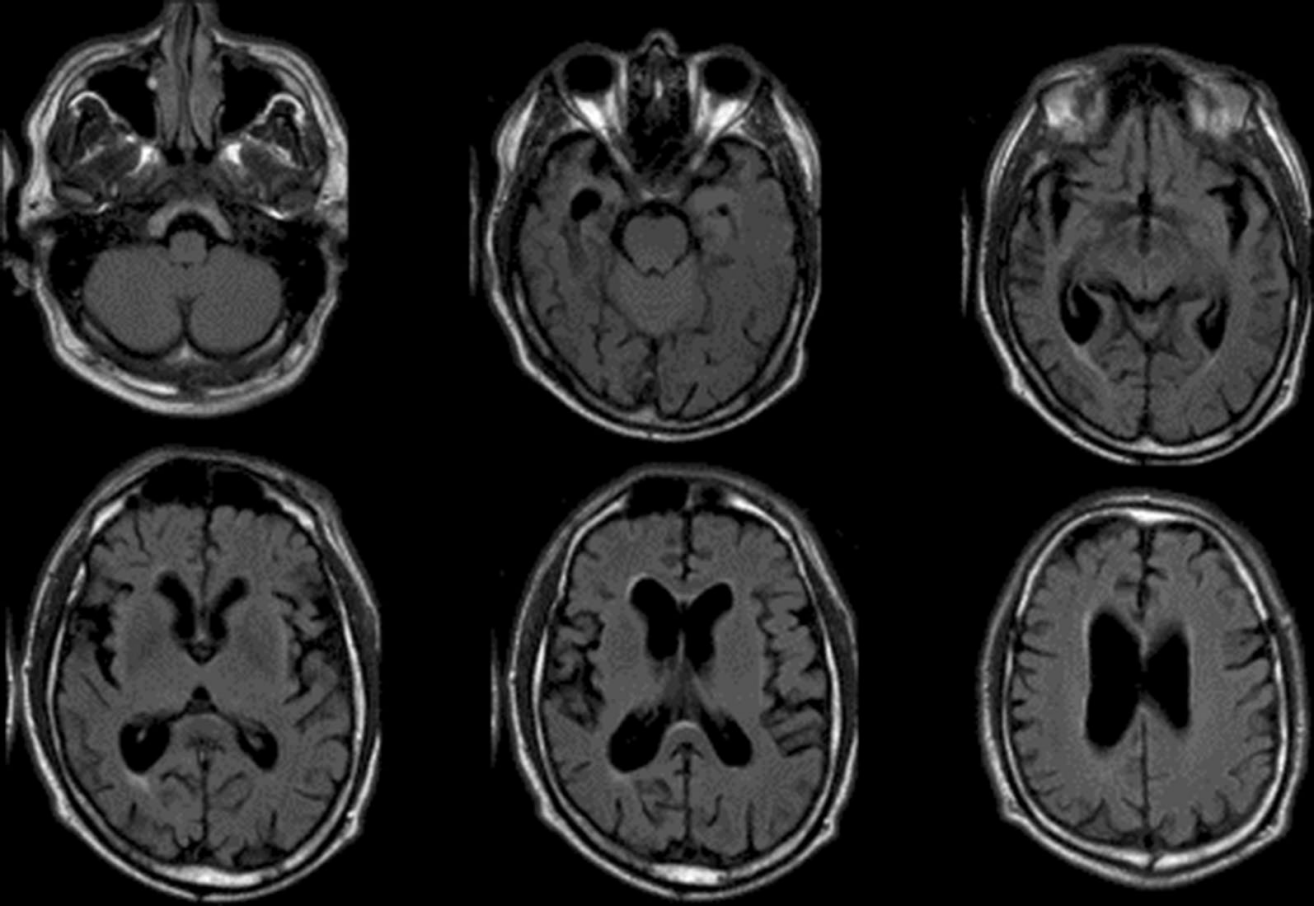
Brain axial fluid-attenuated inversion recovery (FLAIR) magnetic resonance imaging of the patient (42-year-old male) showing asymmetrical brain atrophy, more pronounced in the right hemisphere

**Fig. 2:**
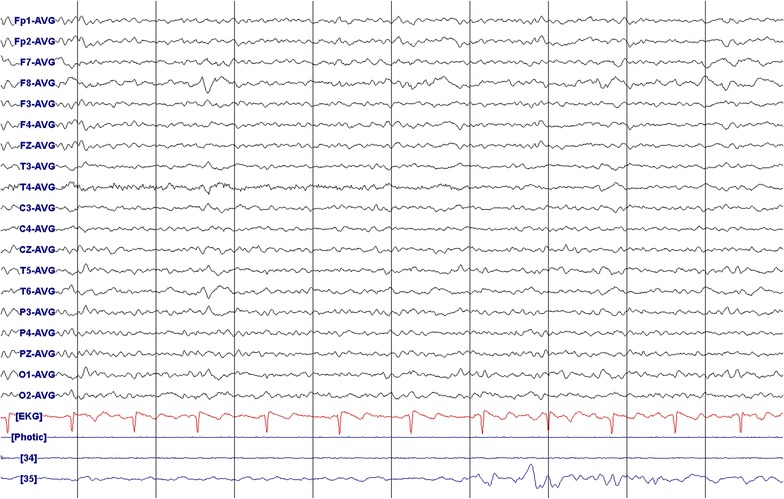
Electroencephalography demonstrating focal slowing and epileptiform discharges in the right fronto-temporal regions of a 42-year old male

Head magnetic resonance imaging (MRI) scan revealed brain atrophy (Fig. 1). There was focal slowing and epileptiform discharges in the right fronto-temporal regions on the electroencephalography (EEG) (Fig. 2).

Blood tests were normal including hepatic function and thyroid tests, although the level of vitamin B12 was slightly decreased (124 pmol/l, reference range 141–489 pmol/l). Human immunodeficiency virus (HIV) 1 and 2 antibodies were negative but the rapid plasma reagin (RPR) test as well as the *T. pallidum* hemagglutination assay (TPHA) were highly positive (RPR 1:32 and TPHA 1:1520) in serum as well as in cerebrospinal fluid (CSF) (RPR 1:8 and TPHA 1:640). CSF showed predominantly lymphocytic pleocytosis (12 cells/mm^3^), the protein was elevated to 0.63 g/l, as well as IgG index (4.31).

Based on clinical pictures and laboratory data, neurosyphilis was diagnosed and intravenous penicillin-G treatment 24 million units per day for 14 days was initiated. Thereafter intramuscular benzathine penicillin of 2.4 million units once per week was injected for 3 weeks. Complementary treatment with divalproex sodium was started as there were epileptiform discharges on the EEG that demonstrated an increased risk for developing of epilepsy, and the patient had emotional problems and agitation.

On the follow-up 6 months later, he had a mild dementia (MMSE 25/30), but there were neither myoclonus nor parkinsonism. Deep tendon reflexes were still brisk but symmetrical. He still had Adie’s tonic pupil in the left and very little constriction to direct light bilaterally. No clinical features of cerebellar dysfunction were detected. Both the serological markers [RPR (1:2) and TPHA] and the above mentioned CSF measures changed to negative.

The patient continued treatment with divalproex sodium 300 mg bid, and enalapril with amlodipine for hypertension. On the subsequent follow-up visits (twice a year), no consistent changes have been found.

## Discussion

Neurosyphilis is a “great imitator”. Its clinical manifestations lack specificity and may mimic several other disorders [3]. In clinical manifestations of meningeal neurosyphilis acute viral meningitis, basal meningitis caused by tuberculosis or meningitis by other microorganism should be considered. Meningovascular syphilis manifests as a stroke and in our case the movement disorder could have been induced by lesion in the midbrain, basal ganglia or cerebellum [3].

The most frequent clinical feature of general paresis is cognitive impairment. Parkinsonism and hyperkinetic manifestations are not often reported in neurosyphilis and the differential diagnosis may be challenging [4, 5]. Psychiatric symptoms are frequent in neurosyphilis and are commonly managed with neuroleptic agents. On the other hand, drug induced movement disorders may develop quite rapidly after initiation of drugs that block dopamine receptors so that the sequentiality might not be detected. In our case report, the patient had not taken any neuroleptics nor other medication that could cause parkinsonism before he was admitted to the hospital. Slightly reduced levels of vitamin B12 is seen frequently in everyday neurologic practice and can hardly explain or contribute to the clinical picture seen in this patient. In addition, movement disorders can be coincidental with infections [4]. Therefore, assessing core criteria of Parkinson’s disease, searching for red flags for atypical parkinsonism and going through all the possible differential diagnosis is important. Furthermore, the prospective follow up of our patient showed improvement in clinical picture after the antibiotic treatment, confirming the causative role of neurosyphilis.

We live in the antibiotic era, but the incidence of syphilis is increasing although no resistance to penicillin has been detected. Neurosyphilis requires 3–4 million units of intravenous aqueous crystalline penicillin G every 4 h for 10–14 days [6, 7]. As some authorities recommend benzathine penicillin 7.2 million units total as three doses intramuscularly after treatment of neurosyphilis, we also continued the treatment with benzathine penicillin 2.4 million units intramuscularly once per week for 3 weeks [2, 6]. The recommended follow-up is every 6 months with CSF examination. Retreatment should be considered if cell count in CSF is not decreased after 6 months or cell count and protein in CSF has not normalised after 2 years [6, 7]. In recent years, men having sex with men has accounted for an increasing proportion of syphilis, and co-infection with HIV has changed its clinical and laboratory profiles, demonstrating a more malignant course and higher antigen titres [2, 8].

The prognosis in early neurosyphilis is quite good and the clinical manifestations of patients with meningeal neurosyphilis or with gummas will usually resolve [6]. In patients with parenchymatous form of neurosyphilis the recovery will not be complete, as in our case.

Neurosyphilis is a treatable cause of dementia and movement disorders, and the disorder must be considered as a possible diagnosis in the routine workup of patients with cognitive decline and movement disorders. In order to achieve the best outcome and avoid a reduction in the patients’ quality of life, an early diagnosis and treatment of neurosyphilis should be applied to prevent an irreversible state of the disease with a poor response to antibiotics [7, 9, 10].

## Conclusions

We present a case of neurosyphilis that manifested with cognitive decline, neuropsychiatric features like irritability and hallucinations, speech disturbances, pupillary defect (tonic pupil in the left, a certain degree of mydriasis with diminished reaction to light), parkinsonism, myoclonus and cerebellar ataxia. This was most likely the cerebral parenchymal form of infection, a clinical type of late-stage syphilis. The patient was treated with penicillin and his symptoms either disappeared (parkinsonism, myoclonus and cerebellar dysfunction) or greatly improved (dementia).

Since syphilis is easily diagnosed and treatable, it should be considered and tested in patients with cognitive impairment and movement disorders. Missing the diagnosis of syphilis is a serious medical mistake that may affect a long-term outcome.

## Additional file

10.1186/s13104-016-2176-2 Video of the patient (42-year-old male) in the pre-treatment phase of neurosyphilis.

## Abbreviations

MMSE: mini mental state examination
MRI: magnetic resonance imaging
FLAIR: fluid-attenuated inversion recovery
EEG: electroencephalography
HIV: human immunodeficiency virus
RPR: rapid plasma regain
TPHA: *Treponema pallidum* hemagglutination assay
CSF: cerebrospinal fluid

## References

[1] Daey Ouwens IM, Koedijk FD, Fiolet AT, van Veen MG, van den Wijngaard KC, Verhoeven WM (2014). Neurosyphilis in the mixed urban-rural community of the Netherlands. Acta Neuropsychiatr.

[2] Jay CA (2006). Treatment of neurosyphilis. Curr Treat Options Neurol.

[3] Chahine LM, Khoriaty RN, Tomford WJ, Hussain MS (2011). The changing face of neurosyphilis. Int J Stroke.

[4] Shah BB, Lang AE (2012). Acquired neurosyphilis presenting as movement disorders. Mov Disord.

[5] Zheng D, Zhou D, Zhao Z, Liu Z, Xiao S, Xing Y (2011). The clinical presentation and imaging manifestation of psychosis and dementia in general paresis: a retrospective study of 116 cases. J Neuropsychiatry Clin Neurosci.

[6] Workowski KA, Berman S (2010). Centers for disease control and prevention (CDC) sexually transmitted diseases treatment guidelines. MMWR Recomm Rep.

[7] French P, Gomberg M, Janier M, Schmidt B, van Voorst Vader P, Young H (2008). European guidelines on the management of syphilis. Int J STD AIDS.

[8] O’Donnell JA, Emery CL (2005). Neurosyphilis: a current review. Curr Infect Dis Rep.

[9] Douglas JM (2009). Penicillin treatment of syphilis: clearing away the shadow on the land. JAMA.

[10] Fenton KA, Breban R, Vardavas R, Okano JT, Martin T, Aral S (2008). Infectious syphilis in high-income settings in the 21st century. Lancet Infect Dis.

